# Mathematics and reading difficulty subtypes: minor phonological influences on mathematics for 5–7-years-old

**DOI:** 10.3389/fpsyg.2015.00221

**Published:** 2015-03-05

**Authors:** Julie A. Jordan, Judith Wylie, Gerry Mulhern

**Affiliations:** School of Education, Queen’s University BelfastBelfast, UK

**Keywords:** subtyping, language, mathematical difficulties, children, longitudinal, reading

## Abstract

Linguistic influences in mathematics have previously been explored through subtyping methodology and by taking advantage of the componential nature of mathematics and variations in language requirements that exist across tasks. The present longitudinal investigation aimed to examine the language requirements of mathematical tasks in young children aged 5–7 years. Initially, 256 children were screened for mathematics and reading difficulties (RDs) using standardized measures. Those scoring at or below the 35th percentile on either dimension were classified as having difficulty. From this screening, 115 children were allocated to each of the mathematical difficulty (MD; *n* = 26), MDRD (*n* = 32), RD (*n* = 22) and typically achieving (*n* = 35) subtypes. These children were tested at four time points, separated by 6 monthly intervals, on a battery of seven mathematical tasks. Growth curve analysis indicated that, in contrast to previous research on older children, young children with MD and MDRD had very similar patterns of development on all mathematical tasks. Overall, the subtype comparisons suggested that language played only a minor mediating role in most tasks, and this was secondary in importance to non-verbal skills. Correlational evidence suggested that children from the different subtypes could have been using different mixes of verbal and non-verbal strategies to solve the mathematical problems.

## INTRODUCTION

A variety of methodologies have shed light on the nature of the relationship between language and mathematics including cross-cultural, correlational, and neuroscientific approaches (e.g., Butterworth, 2008; Dowker et al., 2008). One approach is to compare the mathematics performance of children with different levels of academic achievement, with a focus on subtype differences that mimic the subgroups of children who are grouped in classrooms on the basis of their ability level (e.g., Geary and Hoard, 2001; Koponen et al., 2006; Donlan et al., 2007). In a longitudinal study of children aged 7–9 years adopting both a componential and subtyping approach, Hanich et al. (2001) and Jordan et al. (2003) reported that children with specific mathematical difficulties (MDs) had an advantage over those with comorbid mathematics and reading difficulties (MDRD) in areas where performance may be mediated by language, specifically exact calculation, story problems, and calculation principles. On the other hand, these groups did not differ on tasks reliant on numerical magnitudes, visuo-spatial processing, or automaticity, such as approximate arithmetic. Of course, the curriculum changes as children progress through school and becomes progressively more language dominated, meaning that the relationship between language and mathematics cannot be assumed to be static.

Using a subtyping approach, the present research examined the language requirements of Hanich et al. (2001) and Jordan et al. (2003) mathematical tasks for younger children aged 5–7 years. In contrast to N. Jordan and colleagues’ research on older children, standardized reading tests would not have been suitable for the younger children in the present research. Therefore classifications in the present research were made based on phonological ability, which is strongly associated with early reading progress (Adams, 1990; Ziegler et al., 2010) and with specific language difficulty (e.g., Kamhi and Catts, 1986; Catts et al., 2005). For simplicity, in this paper, the term RD is used to represent both reading difficulty (RD) and phonological difficulty. Inferences about the role of language in mathematics were made by comparing the performance of four subtypes: specific MDs; specific phonological difficulties (RD), comorbid mathematics and phonological difficulties (MDRD) and typical mathematics and phonological achievement (TA). Consistent with Hanich et al. (2001) and Jordan et al. (2003) these subtypes were compared on seven mathematical tasks; namely, exact calculation; story problems, approximate arithmetic, place value, calculation principles, forced retrieval, and written problems.

Hanich et al. (2001) and Jordan et al. (2003) made their conclusions about the language requirements of the tasks based on comparisons between MD and MDRD. They concluded that there was little evidence of MDs amongst RD relative to TA. In contrast, the value of RD/TA comparisons has been demonstrated by Jordan et al. (2010) who found that amongst RD children who did not have MD at age 5 years, approximately half had standardized mathematical ability consistent with MDRD by age 7 years. Closer examination revealed that this was due to the age-related shift in balance from non-verbal to verbal mathematical items in the standardized mathematics achievement test. Indeed, RD made less progress than TA on the more verbal tasks such as number facts, formal calculation, and formal concepts, but had similar growth on tasks with lower language requirements including numbering, number comparison, and informal concepts. As both MD/MDRD and RD/TA subtype comparisons can tell us about the importance of language in mathematical tasks, the present research focuses on both. Further, building upon the work of previous subtyping studies (e.g., Hanich et al., 2001; Jordan et al., 2003), the present research evaluated subtyping as an approach to examining the role of language in mathematics. For this reason the possibility that the relationship between language and mathematical tasks is obscured by subtypes adopting different compensatory strategies is explored. Hereafter follows a synopsis of what is currently known about the language requirements of these seven mathematical tasks.

Exact calculation is an untimed task involving questions such as “how much is 3 plus 5?” or “how much is 6 take away 3?” Previous studies have suggested that language skills are unique predictors of performance on this task (Swanson and Beebe-Frankenberger, 2004; Fuchs et al., 2005, 2006). A longitudinal study examining the mathematical abilities of 5–9-years-old children with specific language impairment (SLI) suggests that these counting-related skills are indeed verbally mediated. The key problem areas identified at age five in these children included producing the number word sequence and counting accurately (Fazio, 1999). Hanich et al. (2001) found that 7-years-old children with MDRD had a more severe impairment in exact calculation than those with MD only. The advantage of MD over MDRD on this task appears to be due to MD’s more accurate use of verbal/finger counting procedures and comparatively better understanding of calculation principles (Jordan and Montani, 1997; Geary et al., 1999; Jordan and Hanich, 2000). Clearly there is strong evidence to suggest this task is verbally demanding for young children, and these effects can be observed from as young as 5 years. Although children with MD were found to outperform MDRD on this task, they still did not perform as well as typically achieving (TA) children at age 7 (Hanich et al., 2001), which is unsurprising given the verbal and non-verbal requirements of counting (Dowker, 2005).

Story problems are untimed arithmetic problems presented in word format that rely on both verbal and non-verbal abilities (Swanson and Beebe-Frankenberger, 2004; Fuchs et al., 2006), and the language requirements of this task are considerably greater than those of exact calculation. Good language skills will help the children to understand the meaning of the story problem, to subsequently form a problem representation, and to read and review the problem rather than relying on holding the problem in memory. Indeed, Jordan et al. (1995) had previously found that children aged 6 with low language ability but adequate spatial ability were impaired on this task relative to normally achieving children. Of course, other non-linguistic skills are also important such as the ability to form concrete or numerical representations of word problems (Dowker, 2005). Subtyping evidence highlights the importance of language ability for this task; comparisons of mathematical subtypes showed that children aged 7–9 years with MDRD consistently perform less well on story problems than those with MD (Hanich et al., 2001; Jordan et al., 2003). Hanich et al. (2001) suggested that, although the performance of MD was weakened by their mathematical deficits, such children may have been able to compensate, to an extent, through their unimpaired verbal skills, and therefore outperform MDRD. Likewise the unimpaired mathematical skills of the RD subtype may have helped alleviate the negative impact of their poor language skills when performing this task. By contrast, the difficulties observed in MDRD, who have weaknesses in both mathematics and reading may have been due their limited compensatory skills. These ideas are speculative and the exact nature of compensatory routes to problem solving is unclear. It is perhaps surprising that the RD subtype did not display a stronger impairment on this task, because understanding the problem through language has been highlighted as a particular area of difficulty for children.

A distinction between approximate (e.g., 2 + 3 = 4 or 11) and exact (e.g., 2 + 3 = ?) arithmetic has been made in educational research (Dowker, 2003). Despite sharing some key skills (e.g., using relations between numbers) and performance on these tasks being associated in young children (Dowker, 1998), discrepancies and dissociations have been found between these tasks in typically developing children (Dowker, 1994, 1998), neuropsychological patients with dyscalculia (Warrington, 1982; Dehaene and Cohen, 1991), and adults with dyslexia (Gobel and Snowling, 2010). Cross-cultural research highlights that cultures that lack number words beyond 5 are able to perform approximate but not exact arithmetic when the problems involve numbers outside their vocabulary range (Pica et al., 2004). Imaging studies show that exact calculation produces greater activation of areas of the brain associated with language, while performing approximate arithmetic leads to greater activation of areas involved in the processing of quantity and spatial information (Dehaene et al., 1999). Subtyping evidence based on 7–9-years-old also indicates that approximate arithmetic has relatively low language demands; both MD and MDRD displayed a similar level of impairment, while RD performed as well as TA (Hanich et al., 2001; Jordan et al., 2003).

Place value tasks assess understanding of how the position of a digit represents a value, as well as ability to name numbers. Children who speak a language with a regular counting system such as Welsh are better at reading two digit numbers than those who speak English which has an irregular counting system (Dowker et al., 2008). Correlational evidence shows that linguistic skills are related to performance on a number naming task, as is spatial span but to a lesser extent than linguistic ability (LeFevre et al., 2010). Subtyping studies indicate that children with MD outperform MDRD on this task (Jordan and Hanich, 2000), and those with RD (Hanich et al., 2001) and SLI (Grauberg, 1998) have difficulty compared to normally achieving children. Contrary to this idea, Hanich et al. (2001) reported that MD and MDRD had a similar level of performance on a place value task. They also found that both MD and MDRD were impaired relative to TA children, concluding that non-verbal skills must also be important. Jordan et al. (2003) found little difference between the subtypes on number naming, suggesting that this part of the task was too easy for children aged 7–9 years, although it is likely that differences will be found in younger children. Overall these findings indicate that both verbal and non-verbal abilities facilitate performance on this task.

Calculation principles such as commutativity, n + 1 and inversion can be used by children to infer the answers to mathematics problems rather than having to fully calculate the answer. Dowker (1998) found that for children aged 5–9 years verbal IQ predicts the use of calculation principles on addition tasks, while both verbal and performance IQ are predictive for subtraction; also predictive of calculation principles use on addition tasks was a verbal/performance IQ discrepancy, possibly because uneven abilities make it difficult to follow standard school-taught procedures, leading children to adopt alternative strategies. Hanich et al. (2001) and Jordan et al. (2003) proposed that when these principles are taught at school, language comprehension may be key to developing a conceptual understanding of them. Subtyping studies have shown that at age 7 children with MD performed at the same level as MDRD; however, by age 9 children with MD significantly outperformed MDRD (Hanich et al., 2001; Jordan et al., 2003).

Fact retrieval assesses the ability to recall answers to problems directly from memory. Subtyping evidence indicates that poor fact retrieval is the most consistent deficit in children with MDs (Russell and Ginsburg, 1984; Geary, 1990, 1993; Geary et al., 1991; Barrouillet et al., 1997; Ostad, 1997, 1998, 1999, 2000; Hanich et al., 2001; Jordan et al., 2003) and in individuals with Turner syndrome who have normal reading ability (Rovet et al., 1994; Molko et al., 2003; Bruandet et al., 2004). These findings strongly indicate that non-verbal factors must influence performance on this task. Although fact retrieval deficits have been identified as a defining feature of MD by many studies, care must be taken when interpreting this finding. As Dowker (2004) points out, arithmetic screening tests often emphasize fact retrieval, consequently it is unsurprising that those children identified as MD on the basis of that test display impairments on a fact retrieval task. While non-verbal skills such as subitizing ability appear to facilitate performance on forced retrieval tasks (Koontz and Berch, 1996), language is also important, as children and adults with specific RDs do not perform as well as normally achieving children on forced retrieval (Geary et al., 2000; Hanich et al., 2001; Simmons and Singleton, 2006; Smedt and Boets, 2010), nor do children with SLIs (Fazio, 1999). There are a number of reasons why children with RDs experience fact retrieval difficulties. For example, Robinson et al. (2002) point out that the repetition method of learning mathematical facts relies very heavily on phonological ability. Additionally, counting is a verbally mediated skill which is commonly used by young children to solve arithmetic problems and correctly solving these problems through counting will strengthen the association between the problem and the solution (Siegler and Shrager, 1984).

Written problems are presented in a vertical visual format and are not read to the children (e.g., Hanich et al., 2001; Jordan et al., 2003). As all problems are displayed in vertical format it is inevitable that some degree of spatial ability is needed for the correct placement and alignment of digits (Dowker, 2005). Evidence suggesting that this task requires good non-verbal skills comes from a study of children with visuo-spatial learning difficulty but normal reading ability (Venneri et al., 2003). Despite performing similar to controls on an oral calculation task, these children displayed impairments on a written calculation task. In addition, Hanich et al. (2001) and Jordan et al. (2003) found that both subtypes with MD had a similar level of impairment on this task, and those with specific RDs did not. This indicates that non-verbal ability plays a greater role than verbal ability in this task. The written problems task used by Jordan et al. (2003) involved problems both with and without a carry/borrow operation. As items with carry/borrow operations are not included in the curriculum for the age group involved in the present study, these items are not included in our adapted version of this task. Relative to normally achieving children, those with visuo-spatial learning difficulty have more difficulty when a carry/borrow operation is required than when it is not (Venneri et al., 2003). Therefore, by removing this requirement, the task makes fewer non-verbal demands and this must be taken into consideration when making predictions about the performance of the subtypes on this task.

Our predictions about the role of language in each of the seven mathematical tasks were made based on studies of older children with MD and what we already know about the normal development of children aged 5–7 years. It is expected that subtyping evidence will indicate that both verbal and non-verbal skills are important for tasks such as exact calculation, story problems, calculation principles, place value, and forced retrieval. On the other hand, performance on tasks such as written problems and approximate arithmetic is likely to involve relatively fewer language skills. In some ways language could play a more important role in task performance in the early years because children aged 5–7 years are more reliant on verbal counting-based procedures than older children (Siegler, 1996). It is possible, however, that as the language skills of the children in the present research will be less well-developed than the sample in Hanich et al. (2001) and Jordan et al. (2003), the TA children will not yet have developed as much of an advantage. Since the maths curriculum becomes progressively more language dominated over the early school years, the relation between language and mathematics cannot be assumed to be static. In this study we explore the consistency of MD and RD relationships in the earliest school years, in children 5–7 years of age.

## MATERIALS AND METHODS

### PARTICIPANTS

The 14 participating schools in this study were from a range of demographic areas, including representation from both urban and rural areas. The Northern Ireland Multiple Deprivation Measure (Northern Ireland Statistics and Research Agency, 2005) rankings for each school’s intake area (1 highest, 890 lowest), indicated that about half of the schools in the sample were located in deprived areas and the other half in the more aﬄuent areas of Northern Ireland (range 2–887).All Year 1 children in the participating schools who had parental consent took part in the screening exercise. The mathematics and phonological difficulty screening tests were individually administered to 256 children with a typical testing session lasting 25–30 min. All participants spoke English as their first language. From this screening, 115 children were retained to allow for comparable sample sizes in the four subtypes of interest (see **Table 1**). At the time of screening the children were aged 5½ years (*M* = 65.59 months; SD = 3.61), and slightly more males (55%) took part than females.

**Table 1:** Subtype ability characteristics and sample sizes.

Subtype	*N*				Mathematical percentile score Mean (SD)	Phonology percentile score Mean (SD)	Verbal percentile score Mean (SD)	Non-verbal percentile score Mean (SD)


	Time							
	1	2	3	4				
MDRD	32	29	30	29	21.34 (9.44)	20.98 (11.57)	22.80 (16.35)	37.72 (23.04)
MD	26	25	25	24	24.42 (10.89)	46.96 (19.21)	42.62 (19.32)	38.81 (27.05)
RD	22	24	19	20	49.27 (14.37)	21.82 (10.76)	31.90 (20.32)	48.62 (25.93)
TA	35	33	29	29	53.57 (16.29)	54.93 (13.63)	46.72 (20.93)	44.63 (23.45)

The specific achievement criteria for each subtype are as follows:

MD: Mathematics score at or below the 35th percentile, and phonological score at or above the 40th percentile.

RD: Phonological score at or below the 35th percentile, and mathematics score at or above the 40th percentile.

MDRD: Both mathematics and phonological scores at or below the 35th percentile.

TA: Both mathematics and phonological scores at or above the 40th percentile.

None: Children with phonological/mathematics scores within the 36th–39th percentile range were unclassified.

### SCREENING MEASURES

Standardized mathematics ability: the Test of Early Mathematics Ability 3, Form A (TEMA 3, Ginsburg and Baroody, 2003) was designed to identify young children with MDs aged 3:0–8:11 years. This test examines formal and informal mathematical skills including number comparison, non-verbal arithmetic, counting, problem solving, numbering skills, numeral literacy, mastery of number facts, calculation skills, and the understanding of concepts. In a study by Mazzocco and Myers (2003) which employed various standardized tests, the Test of Early Mathematics Ability, TEMA-2 (Ginsburg and Baroody, 1990) was reported as the test which produced the most normally distributed data and the greatest stability in test performance over time. The TEMA-3 test has high test–retest reliability (0.95) and correlates moderately (0.55) with the applied problems subtest of the Woodcock–Johnson III Tests of Achievement (Woodcock et al., 2001).

Standardized phonological ability: the Rhyme Detection and Phoneme Deletion (beginning sounds) subtests of the Phonological Abilities Test (PAT; Muter et al., 1997) measure young children’s phonological ability, which is a strong predictor of early reading progress (Adams, 1990). The Rhyme Detection subtest requires a child to select which of three words rhyme with the stimulus word (e.g., cat, which word rhymes?, fish, gun, or hat). For the Phoneme Deletion (beginning sounds) subtest the child is required to delete the first phoneme of a single syllable word (e.g., “bus” without the [b] says [us]).The Rhyme Detection and Phoneme Deletion – Beginning Sounds subtests were selected because overall they are considered to be the best predictors at age 5, 6, and 7 years of scores on the BAS word reading test (Elliott et al., 1997), and they have good test–retest reliability (Phoneme Deletion, 0.84; Rhyme Detection, 0.80).

### VERBAL AND NON-VERBAL ABILITY MEASURES

The Verbal cluster (Word Definitions and Verbal Similarities) and the Non-Verbal subscale (Matrices) of the British Ability Scales 2 (BAS-2; Elliott et al., 1997) were used as ability measures at time 2. In the word definitions test children were presented orally with a word and asked what it meant. In order to be scored as correct, the child had to express the key concepts of the word’s meaning, rather than simply to use it in the correct context. The Verbal Similarities test assesses a child’s ability to explain how two words are similar. For example, when asked why an apple and orange are alike they could say they are both fruits. More general answers that would apply to other categories (e.g., both have skins) are scored as incorrect. The purpose of the matrices subtest is to examine a child’s ability to correctly identify those rules that govern variables in abstract figures. For each item the child must choose which of six alternatives correspond to the geometric pattern that is missing from the matrix. The verbal cluster has a correlation of 0.69 with the corresponding scale of the WISC III, and the non-verbal reasoning cluster has a correlation of 0.56 with the performance scale of the WISC III. All subtests have good internal reliability for 6-years-old (word definitions, 0.79; verbal similarities, 0.88; matrices, 0.78).

### BATTERY OF MATHEMATICAL TASKS

The mathematics test battery comprised seven tasks: exact calculation, story problems, approximate arithmetic, place value, calculation principles, forced retrieval, and written problems. These tasks were closely based on those used previously by N. Jordan and colleagues. with 7–9-years-old. A number of adjustments were made to the tasks so that they would be suitable for children aged 5–7 years. (1) The time limits for approximate arithmetic, calculation principles, and forced retrieval tasks were increased to accommodate the slower processing speeds typical of younger children. (2) The administration time of N. Jordan’s battery was considered too long for young children and therefore the number of items in each task was reduced for the present investigation. (3) Digit correspondence items were omitted from the place value task as they were considered to be too difficult for children aged 5–7 years. (4) Problems with a carry operation were excluded from the written problems task, because this concept is not taught during the early years of primary school. These tasks are described in further detail in Jordan et al. (2009).

### PROCEDURE

**Table 1** displays the ability information for each subtype in the experimental sample, and sample sizes at each time the mathematical test battery was administered. From the 256 children screened, 115 were allocated to the four achievement subtypes and completed the mathematical tasks at time 1. Attrition rates for times 2, 3, and 4 were 3, 10, and 11% respectively. This total sample of 115 included all children identified as having MD or RD. There were too many MDRD and TA children to retain for further longitudinal testing from the 256 children screened. Therefore a subset of children with MDRD was kept; these children were selected carefully to ensure that MDRD were well-matched to MD for mathematics ability and to RD for phonological ability. Similarly, TA children were selected to match the MD group for phonological ability and the RD group for mathematics ability.

All testing was completed on an individual basis at the participating schools by one experimenter who had received police clearance. The study was approved by the School of Psychology Research Ethics Committee at Queen’s University Belfast. The children from the four achievement subtypes were assessed longitudinally on a battery of mathematical tasks from age 5½years onwards. Each child completed the mathematical test battery at four time points separated by 6 monthly intervals, and the administration duration for each session was on average 25 min. Four versions of the battery were constructed in which the order of items was varied for the exact calculation, story problems, approximate arithmetic, and forced retrieval tasks. Each child was given a different version of the test battery at the four time points; the presentation order across the four time points for these versions was varied within each subtype. For all children, the tasks were presented in the following order, (1) exact calculation, (2) story problems, (3) approximate arithmetic, (4) place value, (5) calculation principles, (6) forced retrieval, and (7) written problems. The verbal and non-verbal ability measures were administered at age 6–106 of the 115 (9 were absent) participating children. Testing took 20–30 min depending on the ability level of the child.

## RESULTS

### DATA ANALYSIS PROCEDURES

**FIGURE 1:**
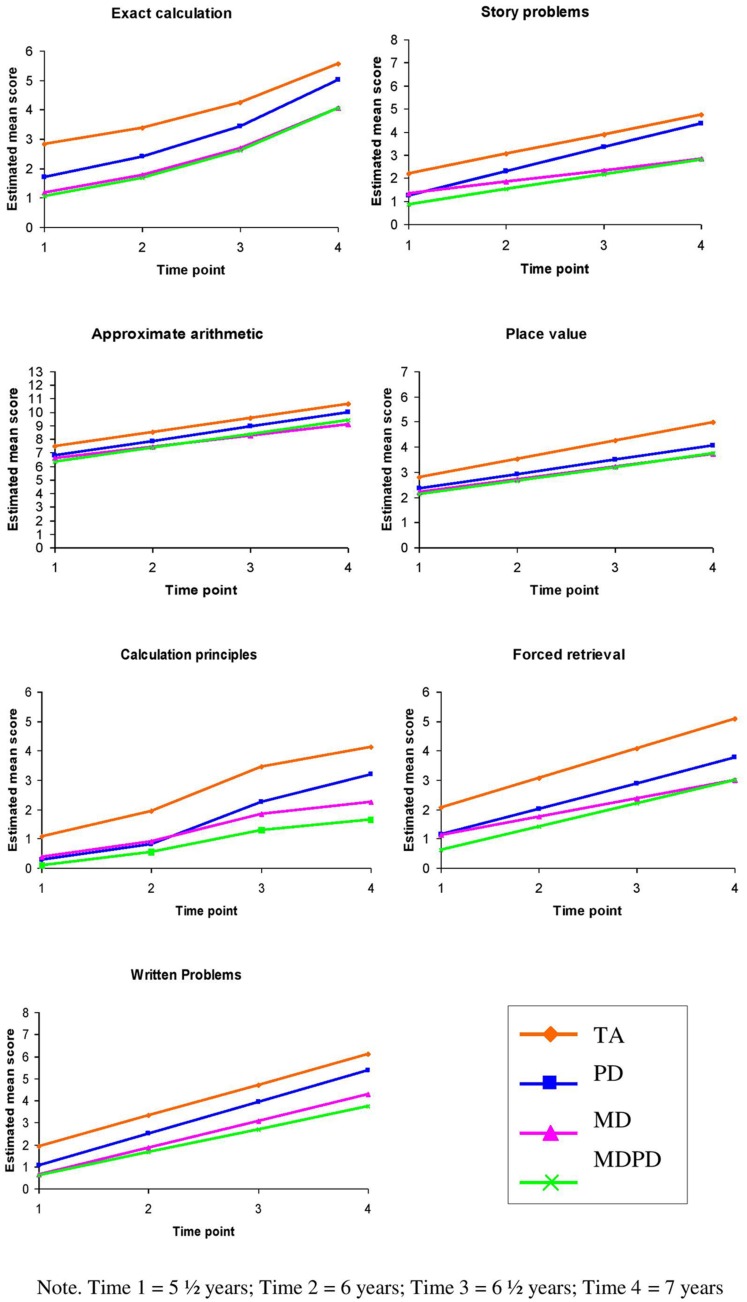
**Estimated mean scores on the mathematical tasks by achievement subtype**.

Raw mean scores and standard deviations are shown in **Table 2**, while estimated trajectories are shown in **Figure 1**. All models were estimated by maximum likelihood (ML) using AMOS 7 (Arbuckle, 2006). Prior to the data analysis, individual and group level growth plots for each of the mathematical subtasks were examined; these provided an indication of the approximate shape of growth for each task. These plots revealed that, for all subtypes, growth appeared to be approximately linear on story problems, approximate arithmetic, place value, forced retrieval and written problems tasks, and curvilinear on exact calculation and calculation principles tasks. It was also apparent that for all tasks there was considerable variation in final status and to a lesser extent growth rates, not only between, but also within, subtypes.

**Table 2:** Mean raw scores and standard deviation on the mathematical tasks by subtype at times 1–4.

Task	Subtype	Time 1		Time 2		Time 3		Time 4	
		Mean	SD	Mean	SD	Mean	SD	Mean	SD
Exact calculation	MDRD	0.81	1.15	1.66	1.63	2.90	1.79	3.76	1.86
	MD	1.62	1.30	3.24	2.03	4.04	1.77	4.63	1.74
	RD	1.82	1.47	3.79	1.89	4.79	1.44	5.20	1.24
	TA	2.74	1.72	4.12	1.73	4.97	1.32	5.59	0.68
Story problems	MDRD	0.84	0.85	1.41	1.09	2.00	1.36	2.72	1.60
	MD	1.46	1.36	2.16	1.57	2.36	1.78	3.33	1.90
	RD	1.14	0.94	2.63	1.50	3.32	1.63	4.25	1.41
	TA	2.17	1.25	2.82	1.74	4.10	1.52	4.66	1.74
Approximate arithmetic	MDRD	5.91	2.43	7.41	2.10	8.23	2.22	8.83	2.07
	MD	6.85	2.39	8.16	2.17	8.44	2.77	9.96	1.78
	RD	7.09	2.64	8.04	2.85	9.16	1.57	10.40	1.85
	TA	7.46	2.23	8.30	2.53	9.31	2.22	10.55	2.03
Place value	MDRD	1.84	0.81	2.45	0.74	2.80	0.76	3.41	0.98
	MD	2.69	0.79	2.84	1.14	3.64	0.91	4.17	0.76
	RD	2.23	0.61	3.00	0.59	3.47	0.70	4.15	0.88
	TA	2.83	1.07	3.55	0.97	4.34	1.26	5.00	1.13
Calculation principles	MDRD	0.13	0.42	0.10	0.41	0.93	1.01	1.55	1.43
	MD	0.42	0.76	0.88	1.09	2.28	1.59	2.54	1.67
	RD	0.32	0.65	1.08	1.06	2.26	1.66	3.55	1.70
	TA	1.11	1.30	1.76	1.44	3.28	1.79	4.00	1.71
Forced retrieval	MDRD	0.63	0.87	0.55	0.83	1.70	1.73	2.59	1.97
	MD	1.19	1.13	2.16	1.65	3.40	1.76	3.58	1.47
	RD	1.23	1.02	2.13	1.54	3.37	1.71	4.10	2.10
	TA	2.00	1.33	3.24	1.73	4.07	1.60	5.07	0.84
Written problems	MDRD	0.31	0.54	1.34	1.54	2.13	2.03	3.31	2.35
	MD	1.08	1.87	2.28	2.03	3.20	2.63	5.25	2.95
	RD	1.18	1.01	3.00	2.36	4.32	2.58	5.35	2.62
	TA	1.77	1.66	3.76	2.45	4.72	2.67	6.00	1.91

*Maximum possible score by task: exact calculation (6), story problems (8) approximate arithmetic (13), place value (7), calculation principles (6); forced retrieval (6); written problems (8).*

Data analysis consisted of two stages, the first of which involved fitting an unconditional model (without predictors) for the whole sample to each of the seven mathematical tasks, to determine if linear or non-linear models provided better fit. In the second stage of the analysis, conditional models were fit to each mathematical task, with achievement group membership as a predictor. Three types of model were tested in this analysis including, linear, freed loading, and quadratic. For all models the slope loading for the fourth time point was set to 0, in order to scale the intercept factor to represent final status. For both linear and non-linear models, the measurement occasions were parameterised in such a way as to reflect rates of growth in terms of 6-months increments.

### LINEAR AND NON-LINEAR UNCONDITIONAL MODEL COMPARISONS

For all tasks, nested model comparisons were used to evaluate whether growth was linear or non-linear. Chi-square difference tests were used to evaluate if the specification of a freed loading model provided a significantly better model fit than a linear model. The results indicated that a non-linear model did not significantly improve model fit for five of the tasks (story problems, approximate arithmetic, place value, forced retrieval, and written problems) suggesting that growth for these tasks was probably linear. By contrast, the chi-square difference test was significant for the exact calculation (χ^2^ = 13.47, df = 2, *p* < 0.01) and for the calculation principles task (χ^2^ = 13.04, df = 2, *p* < 0.01). This would suggest that a non-linear model would better describe the shape of growth for these tasks.

When a quadratic model was run for the calculation principles task multiple estimation problems were encountered, which, according to Bollen and Curran (2006) suggests that this model provides a poor representation of the observed data. In such cases where growth does not follow a strict linear or quadratic trajectory a freed loading model is more suitable, therefore a freed loading model was specified for the calculation principles task. On the other hand, the quadratic model did provide a good fit for the exact calculation task. Although the mean of this factor (χ^2^ = 9.673, df = 1, *p* < 0.01) was significantly different from 0, the variance was not. As there was little variation in acceleration then there would be no value in using achievement subtype membership as a predictor. It would still have been possible to use a quadratic model for this task by fixing the variance; however, to provide more comparability in terms of the interpretation of growth rates across tasks, a freed loading model was also specified for this task.

According to the chi-square test statistics all the models fit well, as there was no significant difference between the models and the data (**Table 3**). The model for story problems and calculation principles do not provide an exact fit according to the root-mean-square error of approximation (RMSEA) statistics; nevertheless, these values are still considered acceptable (Browne and Cudeck, 1993). All models fit well-according to the Tucker Lewis index (TLI) and incremental fit index (IFI) statistics (between 0.9 and 1.2).

**Table 3:** Fit indices for the final unconditional models.

Task	χ^2^	TLI	IFI	RMSEA
Exact calculation	*p* = 0.42	1.01	1.00	0.00
Story problems	*p* = 0.18	0.97	0.98	0.07
Approximate arithmetic	*p* = 0.90	1.16	1.07	0.00
Place value	*p* = 0.63	1.03	1.01	0.00
Calculation principles	*p* = 0.15	0.94	0.98	0.08
Forced retrieval	*p* = 0.58	1.02	1.01	0.00
Written problems	*p* = 0.66	1.03	1.01	0.00

*Fit indices Ideal fit; Chi-square test statistic (χ^*2*^) = non-significant *p*-value; Tucker-Lewis index (TLI) = 1; Incremental fit index (IFI) = 1; Root-mean-square error of approximation (RMSEA) ≤0.05.*

**Table 4** displays the means and variances for final status and the growth rates for the combined sample on each task. For all tasks the variances for the growth rates and final status were significantly greater than zero, therefore the analysis of parameter correlates could be pursued. In the next stage of data analysis, achievement subtype was added as a predictor to the model for each task.

**Table 4:** Estimated parameters for the combined sample by task.

Task	Final status		Growth rate		Covariance (FS/GR)


	Mean	Variance	Mean	Variance	
Exact calculation	4.71	2.19	0.98	0.25	0.36
Story problems	3.72	2.87	0.75	0.16	0.58
Approximate arithmetic	9.83	2.92	0.99	0.36	0.70
Place value	4.17	1.01	0.59	0.09	0.23
Calculation principles	2.82	2.83	0.77	0.24	0.73
Forced retrieval	3.76	3.18	0.83	0.29	0.76
Written problems	4.89	5.33	1.26	0.43	1.21

*FS/GR is final status/growth rate. All significant at the *p* < 0.05 level.*

### CONDITIONAL MODELS WITH ACHIEVEMENT GROUP MEMBERSHIP AS A PREDICTOR

To enable between-group comparisons, final status and growth rates were regressed on three dummy variables. In the first set of models, MD, RD, and TA were coded as 1 and MDRD, the reference group, was coded as 0. In order to compare all groups, models were also estimated with TA and then with RD as the reference group.

The fit indices (**Table 5**), show that most models still fit well after the predictor was added and the model fit actually improved for the story problems and calculation principles tasks. The fit indices for the approximate arithmetic task model are not as good as they were before achievement subtype was added to the model; despite this the overall model fit for this task is still acceptable.

**Table 5:** Fit indices for the conditional models.

Task	χ^2^	TLI	IFI	RMSEA
Exact calculation	*p* = 0.454	1.00	1.00	0.00
Story Problems	*p* = 0.193	0.96	0.99	0.05
Approximate arithmetic	*p* = 0.072	0.85	0.95	0.08
Place value	*p* = 0.322	0.98	0.99	0.04
Calculation principles	*p* = 0.423	1.00	1.00	0.01
Forced retrieval	*p* = 0.258	0.97	1.00	0.05
Written problems	*p* = 0.287	0.97	0.99	0.04

For all tasks, there was significant variation in final status which was unexplained by achievement subtype membership (**Table 6**). With the exception of story problems, after controlling for achievement subtype membership, there was still considerable unexplained variance in growth rates. In fact, for all tasks, achievement subtype membership explained much less of the variance in growth rate than in final status. Achievement subtype membership explained much more variance in the growth rates for story problems (24%) and calculation principles (19%) than for the other mathematical tasks. From the remaining tasks, approximate arithmetic is the one for which achievement subtype membership explains the least variance, both in terms of final status (12%) and growth rates (2%). It is likely that, for these reasons, the model for the approximate arithmetic task fits less well after achievement subtype membership was added as a predictor to the model.

**Table 6:** Variance explained by achievement subtype membership.

Task	Final status		Growth rate	
	Variance	*R*^2^	Variance	*R*^2^
Exact calculation	1.72**	0.20	0.23**	0.02
Story problems	2.03**	0.29	0.12	0.24
Approximate arithmetic	2.59**	0.12	0.38*	0.02
Place value	0.73**	0.29	0.09**	0.08
Calculation principles	1.84**	0.36	0.21**	0.19
Forced retrieval	2.37**	0.26	0.26**	0.07
Written problems	4.28**	0.18	0.36**	0.07


Variance refers to the variance in intercepts and slopes remaining after controlling for achievement subtype membership. *R*^*2*^ the amount of variance in the model explained by achievement subtype membership. * *p* < 0.05, ** *p* < 0.01.

Growth curve model comparisons between the MD and MDRD subtypes revealed no significant differences in terms of final status and growth rates on any of the mathematical tasks (**Table 7** and **Figure 1**). Furthermore, both subtypes had significantly lower final status on all tasks relative to TA children. The MD subtype displayed significantly weaker growth over the 18 months period than normally achieving children on the story problems, place value, calculation principles and forced retrieval tasks. Despite MDRD and MD having similar growth rates across tasks, the only task on which MDRD experienced significantly less growth than normally achieving children was calculation principles.

**Table 7:** Estimated final status (age 7 years) and growth rates by achievement subtype.

	MDRD		MD		RD		TA	
	FS	GR	FS	GR	FS	GR	FS	GR
Exact calculation	4.07^a,b^	1.00	4.06^a,b^	0.96	5.02	1.10	5.56	0.91
Story problems	2.80^a,b^	0.64^b^	2.82^a,b^	0.50^a,b^	4.38	1.05	4.74	0.84
Approximate arithmetic	9.39^a^	1.01	9.12^a^	0.84	10.00	1.06	10.63	1.05
Place value	3.74^a^	0.54	3.72^a^	0.50^a^	4.06^a^	0.57	4.98	0.73
Calculation principles	1.66^a,b^	0.52^a,b^	2.26^a^	0.63^a,b^	3.19^a^	0.97	4.13	1.02
Forced retrieval	2.99^a^	0.79	2.99^a^	0.62^a^	3.76^a^	0.87	5.09	1.01
Written problems	3.73^a^	1.04	4.30^a^	1.22	5.39	1.44	6.10	1.39

*FS (final status), GR (growth rate). Significant differences, *p* < 0.05. ^a^TA > MDRD, MD, RD; ^b^RD > MDRD, MD.*

The RD subtype had significantly greater final status than both MD and MDRD on the exact calculation and story problems tasks and only the MDRD subtype on calculation principles. On the story problems and calculation principles tasks the RD subtype had significantly greater growth than both the MD and MDRD subtypes.

Children with specific RDs performed less well than normally achieving children at time 4 on all tasks; these differences were significant for place value, calculation principles, and forced retrieval. Despite these differences, RD and TA had comparable growth rates across all tasks. Ceiling effects were apparent on exact calculation and forced retrieval for the normally achieving subtype at the end of the developmental period under investigation. Consequently, these effects may have impeded our ability to detect significant differences between the subtypes with learning difficulties and the TA subtype in terms of final status and growth rate on these tasks. Based on the estimated scores produced by the growth curve analysis, overall the consistent pattern for all tasks (**Figure 1**) was: TA outperformed RD, and MD and MDRD had a similar level of impairment relative to RD and TA.

### RELATIONSHIPS BETWEEN VERBAL, NON-VERBAL ABILITY, AND THE MATHEMATICAL TASKS

The relationship between verbal, non-verbal and phonological ability and performance on each of the mathematical tasks (time 4) was investigated using Pearson product-moment correlations. Scores on the ability measures were correlated with performance on each mathematical task to examine the relationship between these abilities in TA children and in the subtypes with learning difficulties (**Table 8**).

**Table 8:** Correlations between phonological, verbal and non-verbal ability, and performance on each mathematical task by subtype.

Subtype	Task	Phonological ability	Verbal ability	Non-verbal ability
TA	Exact calculation	0.11	–0.31*	–0.03
	Story problems	0.12	0.43*	0.23
	Approximate arithmetic	0.34*	0.17	–0.03
	Place value	0.41*	0.40*	0.40*
	Calculation principles	0.04	0.15	0.29
	Forced retrieval	0.05	–0.09	0.11
	Written problems	0.40*	–0.01	0.05
RD	Exact calculation	–0.25	–0.15	0.33
	Story problems	0.03	0.10	0.46*
	Approximate arithmetic	–0.12	0.19	0.60*
	Place value	–0.26	0.15	0.42*
	Calculation principles	0.01	0.32	0.46*
	Forced retrieval	–0.01	–0.21	0.43*
	Written problems	–0.14	–0.03	0.46*
MD	Exact calculation	0.78*	0.03	0.38*
	Story problems	0.76*	0.39*	0.38*
	Approximate arithmetic	0.63*	–0.03	0.13
	Place value	0.59*	0.21	0.22
	Calculation principles	0.46*	0.17	0.33
	Forced retrieval	0.87*	0.13	0.28
	Written problems	0.72*	0.22	0.23
MDRD	Exact calculation	0.21	–0.06	0.37*
	Story problems	0.40*	–0.06	0.34*
	Approximate arithmetic	0.19	0.15	–0.05
	Place value	0.18	0.02	0.36*
	Calculation principles	0.16	0.28	0.07
	Forced retrieval	0.28	–0.07	0.58*
	Written problems	0.10	–0.06	0.34*

*p < 0.05.

## DISCUSSION

The present research examined the role of phonological ability in the mathematical development of 5–7-years-old using a subtyping approach. Contrary to Hanich et al. (2001) and Jordan et al. (2003), both MD and MDRD children aged 5–7 years in the present study exhibited very similar performance across all mathematical tasks, as evidenced by their final status (age 7 years) and growth rates. Despite initial matching for mathematics ability with TA, RD had consistently weaker performance on place value, calculation principles, and forced retrieval, suggesting that phonological ability is important for children aged 5–7 years when performing these particular tasks. In addition to age-related differences, some of the adaptations made to N. Jordan’s original battery of tasks may have led to minor qualitative differences in the nature of the tasks, possibly limiting comparability with the present investigation. Furthermore, the use of different mathematics and RD screening may partly explain the differences in findings between the present research and that of Hanich et al. (2001) and Jordan et al. (2003). While phonological ability is related to both language and reading ability, as Robinson et al. (2002) point out, phonological ability may directly influence mathematics achievement. For example, the repetition method of learning mathematical facts relies very heavily on phonological ability. As each number fact is repeated phonological information must be both generated and stored and each repetition strengthens the association between the problem and the answer. The greater the association between the answer and the problem the greater the chance of successful recall. This may explain why children with poor phonological ability but strong non-verbal abilities were more impaired in the present research compared to children with specific RD in other research (Hanich et al., 2001; Jordan et al., 2003).

As MD and MDRD were initially matched for mathematics ability, it was not expected that MDRD would perform worse than MD on all tasks. Rather it was expected that MDRD would have weaker performance than MD on tasks with stronger language requirements, and have similar or possibly better results than MD on tasks with fewer language requirements if they could adopt effective compensatory strategies. Despite a body of research showing that language plays a key role in many of the mathematical tasks, the MD and MDRD subtypes performed similarly on all tasks. It is difficult to explain why RD performed worse than TA on some tasks, yet MDRD and MD had similar performance despite having different phonological abilities. Of course not all skills associated with mathematics were assessed in this study and it is possible that MDRD were able to achieve comparable performance to MD through the use of alternative skills. Indeed, uncertainty exists over the exact number of deficits that may contribute to children’s MDs (Swanson, 2007) and to what extent these occur in isolation or co-occur in various combinations. To date, numerous deficits have been linked to MD, including poor number sense (Butterworth, 1999), visuo-spatial difficulties (Rourke and Conway, 1997) and executive dysfunction (Geary et al., 2007a) and as a group the MDRD subtype may have had superior skills to MD in any of these areas.

The possibility that these subtypes were relying on different strategies when completing the different mathematical tasks has previously been suggested (Hanich et al., 2001). While this is a somewhat speculative suggestion, a correlational analysis performed in the present research does lend support to this idea. Phonological ability was consistently highly associated with the performance of MD on each of the mathematical tasks, whereas non-verbal and verbal ability were not. It may seem surprising that phonological ability was related to maths performance much more than verbal ability despite both being language-based tasks. However, compared to the verbal IQ tasks used in the present study, the phonological tasks require very basic skills, for example, rhyming and the ability to break words down into phonemes (Muter et al., 1997). In contrast, the verbal subtests of the British Ability Scales require a broad range of higher order skills such as vocabulary knowledge, reasoning, and abstract thinking (Elliott et al., 1997). By contrast only non-verbal ability predicted the performance of the RD subtype on each of the mathematical tasks. Similarly, non-verbal ability was a better predictor than verbal ability of MDRD children’s performance on most tasks. These findings suggest that the children with MD may tend to use their intact verbal skills more often than their impaired non-verbal skills to solve problems. On the other hand, the RD subtype may use their intact non-verbal skills more than their weak verbal skills to solve problems. These findings indicate that language does not play a ‘standard’ role in mathematical tasks, rather the role of language will vary from individual to individual depending on their particular strengths and weaknesses. Indeed, cross-cultural evidence shows that amongst cultures where counting words are not available, children solve non-verbal calculation problems using spatial strategies. In contrast English-speaking children hardly ever use spatial strategies and tend to rely more on counting words (Butterworth et al., 2011).

Greater knowledge of individual differences in strategy use would allow interventionists to design interventions based on the strength and weaknesses of the child (Dowker and Sigley, 2010) rather than forcing them to use ‘standard procedures’ which may not suit their learning style. For example, students with specific RD often have difficulty recalling number facts (e.g., Simmons and Singleton, 2006; Smedt and Boets, 2010), and for these students use of derived strategies based on facts that they can recall may be more appropriate. In some cases students will need assistance to develop appropriate strategies and in other cases they may come up with their own strategies. For example, university students with specific RDs mention developing their own visual strategies (e.g., diagrams) to understand and solve mathematical problems and to compensate for their relatively weak verbal skills (Perkin and Croft, 2007). There has been some research on how children with uneven abilities solve exact calculation compared to TA children (e.g., Geary et al., 2000; Jordan et al., 2003; Wylie et al., 2012). Generally speaking these studies show that children with MD and MDRD employ a different strategy mix to RD or TA when solving problems, either by relying on developmentally immature strategies or trying to use mature counting strategies before developmentally ready. However, less is known about the use of individual strategies on other mathematical tasks (e.g., place value, geometry). In addition, asking children about how they solve problems can only identify different procedures, it does not tell us about individual differences in terms of how children represent number in the brain. While much is now known about the neural basis of numerical cognition (Butterworth and Walsh, 2011), less is known about how children with uneven abilities represent mathematical problems at a neural level compared to TA children.

The performance of TA on each of the tasks was correlated with phonological, verbal and non-verbal ability, to indicate the language and non-verbal requirements of these tasks for children with good verbal and non-verbal skills who are more likely to follow standard procedures. For TA children, the correlation analyses did not highlight any clear bias towards verbal or non-verbal strategy use. In contrast to previous research (Dowker, 1998), verbal ability did not predict the performance of TA children on most mathematical tasks. It could be the case that as children get older and their verbal skills develop further they are better able to utilize these skills when solving mathematics problems. If so, this may partially account for the stronger relationship between maths and verbal IQ observed in Dowker’s sample which comprised children aged 5–9 years. It was surprising that for TA verbal and non-verbal ability did not relate more consistently with the mathematical tasks; however, the correlations may have been weakened by ceiling effects on the mathematical tasks.

A key aim of the present research was to evaluate the suitability of subtyping as an approach to examining the role of language in mathematics. On a positive note, subtyping has greater ecological validity than correlational analyses, in the sense that children are arbitrarily classed as having MD in the classroom. Indeed, decisions regarding whether or not to intervene are often made based on these arbitrary cut-off points. However, in contrast to correlational approaches, subtyping does not use full variation in statistical analysis. It is important to note that a key limitation of the present study and the previous work of Hanich et al. (2001) and Jordan et al. (2003), was the use of subtyping classification based on an assessment at a single time point. Research on subtype stability has shown that while some young children have persistent MDs, others have a more variable pattern of achievement and can be mislabeled if assessed only once (Mazzocco and Myers, 2003). It is possible that the lenient cut-off point (35th percentile) used in the present analysis may have affected the results. Indeed, Geary et al. (2007b) found that children with mathematical disabilities (<15th percentile) and those with low maths achievement (23rd–39th percentile) displayed qualitatively different profiles of deficit. However, Jordan and Hanich (2003) found that children with below average (<15th percentile) and those with low (15th–30th percentile) mathematics achievement displayed qualitatively similar performance on a range of mathematical tasks.

The present analysis has identified a further limitation of using a subtyping approach. Assessing the language requirements of these tasks based on subtyping comparisons is difficult because in the present study, and in (Hanich et al.’s (2001) investigation, on some occasions the RD subtype was significantly impaired, yet the MDRD subtype performed at a similar level to the MD subtype. The opposite situation was also observed by Hanich et al. (2001), where the MD subtype significantly outperformed the MDRD subtype yet the RD subtype was not significantly impaired. These inconsistencies indicate that subtyping on its own as a methodology does not give a good indication of the verbal/non-verbal requirements of a task. Indeed, Bartelet et al. (2014) have concluded that it is difficult to draw conclusions from subtyping evidence alone due to the heterogeneous nature of MD. Despite these limitations, subtyping in conjunction with correlational evidence does provide important insights into the role of language in mathematics. The findings from the present study suggest that children can achieve very similar performance levels via different mixes of verbal and non-verbal strategies. Consistent with the existing body of research on mathematical tasks (e.g., Dowker, 2005; Dowker et al., 2008; LeFevre et al., 2010), subtypes with weak verbal or non-verbal ability do not perform as well as their typically achieving counterparts, suggesting that both language and non-verbal skills are important in achieving age-appropriate performance on most tasks.

## Conflict of Interest Statement

The authors declare that the research was conducted in the absence of any commercial or financial relationships that could be construed as a potential conflict of interest.
